# Comparative effects of open-skill and closed-skill sports on executive function in university students: a 16-week quasi-experimental study

**DOI:** 10.3389/fpsyg.2024.1457449

**Published:** 2024-10-07

**Authors:** Yu-fan Li, Tianyu Gao, Li-peng Luo, Shan He

**Affiliations:** ^1^School of Outdoor Sports, Guilin Tourism University, Guilin, China; ^2^School of Physical Education, Jinan University, Guangzhou, China; ^3^Department of Health and Physical Education, The Education University of Hong Kong, Hong Kong, Hong Kong SAR, China

**Keywords:** soccer, cognition, cognitive function, intervention, golf

## Abstract

**Introduction:**

Previous reviews have often concluded that open-skill sports are more effective at enhancing executive function (EF) than closed-skill sports. However, this conclusion may not hold for closed-skill sports with high cognitive demands, such as golf. This study aimed to compare the effects of football (open-skill) and golf (closed-skill) training on enhancing EF in university students.

**Method:**

Using a quasi-experimental, pre-post test design, 63 male participants were assigned to three groups: football (*n* = 21), golf (*n* = 21), and a sedentary control group (*n* = 21). Over 16 weeks of training, the intervention groups engaged in four 90-min training sessions per week, while the control group attended one 80-min physical education class per week. Assessments were conducted before and after the intervention. EFs, including inhibition control and working memory, were assessed using the Flanker task and Corsi-block tapping task, respectively. Cardiovascular fitness (CRF) was measured by the multi-stage fitness test.

**Results:**

The golf group showed significant improvements in inhibition control from pre- to post-intervention (*p* = 0.02, *d* = 0.26), while the football and control groups did not exhibit significant changes. Post-intervention comparisons indicated no significant differences in EF performance between the golf and football groups; however, both outperformed the control group (golf, *p* = 0.002, *d* = 0.99; football, *p* = 0.01, *d* = 0.67). No significant improvement was observed in working memory for any group. Additionally, changes in CRF were not significantly correlated with EF performance.

**Conclusion:**

This study provides preliminary evidence that golf, a closed-skill sport with high cognitive demands, can effectively improve inhibitory control after 16 weeks of training. This improvement is comparable to that observed in football, an open-skill sport. The findings also suggest that the cognitive demands of the sports, rather than improvements in physical fitness, may be primarily responsible for the enhancements in EF.

## 1 Introduction

Executive function (EF) encompasses critical cognitive processes such as information updating, planning, attention, working memory, and problem-solving, all of which are vital for goal-directed behavior and adapting to environmental changes (Diamond, 2013). The COVID-19 pandemic has exacerbated cognitive impairments, including cognitive decline and ‘brain fog’, underscoring the urgent need for effective strategies to enhance EF (Altuna et al., 2021). Exercise has emerged as a promising intervention to improve EF (De Sousa et al., 2021; Teo and Goodwill, 2022), as evidenced by review studies indicating that various exercise regimens can significantly enhance inhibitory control and working memory in youth, although effect sizes are typically low to moderate (see review, Liu et al., 2020).

While the general benefits of exercise on EF are recognized, the nuanced relationships and underlying mechanisms, especially regarding different types of exercise and EF, remain not well understood (Logan et al., 2023). Studies suggest that the type of cognitive load inherent in different sports may play a critical role in determining their impact on EF (Fuster et al., 2021; Yongtawee et al., 2022). For instance, open-skill sports, such as football, which require constant adaptation to unpredictable environments, may enhance specific aspects of EF differently compared to closed-skill sports like swimming, which involve more predictable and self-paced environments with minimal external variability (Zhu et al., 2020; Russo et al., 2022). Athletes in open-skill sports exhibit more variability in their motor responses, as they must adapt to the specific environmental conditions of each situation. In contrast, closed-skill athletes typically follow set patterns in their motor responses, resulting in greater consistency (Zhu et al., 2020; Russo et al., 2022). This distinction contributes to differences in EF performance between closed-skill athletes and open-skill athletes. Specifically, athletes in open-skill sports have shown superior sustained attention (Ballester et al., 2018), inhibitory control (Wang et al., 2013, 2017), and cognitive flexibility (Yu et al., 2017) compared to athletes in closed-skill sports and non-athletes (Yongtawee et al., 2021). A randomized control trial by Tsai et al. (2017) further supports this distinction, demonstrating that after 6 months of intervention, novel elderly male participants in open-skill exercises (i.e., table tennis) improved reaction times in task-switching tasks, while closed-skill exercises (i.e., bike riding and brisk walking/jogging) enhanced accuracy in memory tasks.

Neuroimaging studies provide further insight into the neurocognitive effects of different sports. Studies using EEG or fMRI have observed that open-skill sports are associated with increased neural efficiency and enhanced connectivity in brain regions related to EF during cognitive tasks (Takahashi and Grove, 2023), and are generally thought to provide greater benefits to EF than closed-skill sports (Wang et al., 2017; Gu et al., 2019; Zhu et al., 2020; Heilmann et al., 2022). However, existing comparisons between open- and closed-skill sports have not sufficiently clarified whether closed-skill exercises with high cognitive demands could lead to similar improvements in EF as open-skill exercises. Most previous studies have focused on closed-skill exercises like running or swimming, which involve minimal cognitive load (see reviews, Gu et al., 2019; Zhu et al., 2020; Heilmann et al., 2022). A closed-skill sport with high cognitive demands, such as golf, might yield different results.

Golf, a closed-skill sport, integrates physical, sensory, cognitive, and social components (Murray et al., 2017; Shimada et al., 2018). Mastering the golf swing requires high levels of hand-eye coordination, static postural control, and sensorimotor control. During a game, strategic planning, information management, and adapting to changing environmental conditions highlight the cognitive demands of golf (Jäncke et al., 2009; Shimada et al., 2018). Studies have examined golf’s impact on various aspects of EF, such as attention, inhibition control, working memory, and cognitive feasibility (Gallicchio et al., 2017; Shimada et al., 2018; Roberts et al., 2021; Wang et al., 2020). Currently, most studies focus on its role in enhancing EF and delaying cognitive decline in the elderly. For example, Shimada et al. (2018) found that a 24-week golf training program significantly improved both immediate and delayed logical memory in older adults. Similarly, Stroehlein et al. (2021) reported that elderly individuals with subjective memory complaints who completed a 22-week golf training program performed better in inhibition tasks than a waiting list control group. Bezzola et al. (2011) observed that 40 h of golf practice, conducted as a leisure activity with individualized training, increased grey matter in task-relevant cortical networks involving sensorimotor regions in older adults. Limited studies on younger populations, such as that by Kim et al. (2015), revealed that professional golfers exhibited greater functional connectivity between the cerebellum and frontal lobes compared to non-golfers, indicating enhanced brain functions related to motor control and inhibition. These promising findings warrant further exploration into whether golf can improve EF as effectively as open-skill sports in younger populations.

Football, an open-skill sport, requires continuous adaptation to changing environments, influencing cognitive processes differently than closed-skill sports. Studies indicate that EF significantly influences football performance, correlating with better outcomes in goals and assists, and cognitive abilities can predict performance levels (Vestberg et al., 2017; Sakamoto et al., 2018). Football-related training programs have demonstrated both acute and chronic positive effects on EF. For instance, Won et al. (2017) found that a single session of indoor football among college-aged players significantly impacted brain networks responsible for attention allocation and classification speed during inhibitory control tasks. Dong et al. (2024) reported that 70 sessions of 30-min football juggling over 82 days enhanced young adults’ EF performance, particularly in inhibition and shifting, by increasing functional connectivity within the frontal, temporal, and cerebellar regions. Additionally, Xiao et al. (2024) found that a specific football training program improved working memory in adolescents with intellectual disabilities by enhancing activation in the right frontopolar area of the brain.

The mechanisms through which exercise influences EF performance are multifaceted and complex. Cardiovascular fitness (CRF), in particular, has been strongly correlated with EF improvements across numerous studies (e.g., Mekari et al., 2019; Aghjayan et al., 2021; Logan et al., 2023). Football, which combines both aerobic and anaerobic exercise, may be more effective at enhancing CRF compared to golf, suggesting that football could offer greater benefits to EF through improved CRF. However, cognitive training embedded within physical exercise regimens has also been exceptionally effective in enhancing EF (Biazus-Sehn et al., 2020; Gavelin et al., 2021). Although golf typically involves lower exercise intensity, it includes periods of varying intensity and significant cognitive demands (Murray et al., 2017; Shimada et al., 2018), which may render it equally effective as football in improving EF. Given that the relationship between CRF and EF becomes more pronounced after early adulthood (Scisco et al., 2008), university students, who are at a critical stage of EF development, are an ideal group to study the effects of different types of exercise on EF. Improvements in EF during this period can have a significant impact on academic performance, decision-making and overall cognitive health (Diamond, 2013).

To better understand how different forms of exercise impact EF performance, this study aims to compare the effects of football and golf on EF in male university students. Based on the previous literature, we hypothesized that: (1) both football and golf would be effective in enhancing EF compared to a non-exercise group; and (2) the effectiveness of football and golf in enhancing EF would be equivalent.

## 2 Method

### 2.1 Participants

Sixty-three male university students were recruited using a convenience sampling strategy. Participants were recruited from the first author’s university through advertisements posted on campus. Interested students called the office number listed on the poster and left personal information such as name, contact information, age, and major. Those who met the inclusion criteria were invited to participate, with enrollment continuing until the quota was reached.

Participants were assigned to one of three groups: golf (*n* = 21), football (*n* = 21), and a sedentary control group (*n* = 21). The two intervention groups comprised physical education students with comparable physical fitness scores. After providing consent, participants voluntarily joined either the golf or football group to receive specific training. The control group consisted of students from normal academic classes who exhibited sedentary behavior. Participants’ demographic information is listed in Table 1.

**Table 1:** Demographic information.

Groups	Golf	Football	Control
Age (years)	20.95 (1.02)	20.81 (0.98)	20.62 (0.87)
Heart rate (/min)	65.33 (6.191)	63.62 (5.4)	73.43 (6.49)
BMI	23.1 (1.73)	22.56 (1.55)	24.04 (2.63)
IPAQ (METS)	4650.57 (837.3)	3932.71 (903.79)	1399.81 (506.88)

Data are presented as mean (SD). BMI indicates the body mass index. IPAQ indicates the general physical activity level out of training over the last 7 days during the intervention.

Eligibility criteria included no history of injury within 1 month prior to the study, no medication use, no special education needs, and being predominantly right-handed. Additionally, participants were required not to have contracted COVID-19 within the past 3 months. Students completed the International Physical Activity Questionnaire (IPAQ), and only those who reported sitting for at least 540 min per weekday were included in the control group (Bauman et al., 2011; Liang et al., 2024; Scholes et al., 2016).

This study was approved by Guilin Tourism university’s research board [Ref. 桂旅科研 (2023)-68] and adhered to the principles outlined in the Declaration of Helsinki. All participants were informed of the experimental procedures and potential risks and voluntarily signed consent forms. No participants dropped out of the study, and no financial incentives were provided.

An *a priori* power analysis was performed using G*Power (version 3.1.9; Faul et al., 2007) to determine the minimum sample size needed to achieve a power level of 0.8. This analysis was based on data from a previous study that involved 20 participants per group (Ballester et al., 2018), comparing performance on the psychomotor vigilance task among adolescent football athletes, track and field athletes, and age-matched non-athlete controls. Accounting for a 5% dropout rate, a total of 63 individuals were required.

### 2.2 Design and procedure

A single-blinded, quasi-experimental pre-posttest design was employed. Two trained assessors, blinded to the research aims, conducted all assessments and did not participate in the intervention. Baseline (pre-test) assessments were conducted at the beginning of the academic year, starting on September 4 and lasting for 2 weeks. These assessments included measurements of demographic information, CRF, and EFs such as inhibition control and working memory. Participants then underwent a 16-week intervention starting on September 19, involving golf, football, or control (i.e., physical education classes) based on their group assignment. Post-intervention assessments of CRF and EFs were conducted on January 8 of the following year. To account for other physical activities outside of class that might affect EF performance at post-test, participants were asked to report their daily physical activity levels.

On testing days, participants were prohibited from consuming beverages containing caffeine or alcohol and from engaging in any strenuous physical activities for the 24 h prior to visiting the lab. They were instructed to maintain a consistent diet in the days and hours before the testing day to minimize variations from baseline tests. This ensured that test results were not affected by participants’ nutritional status.

The pre-test procedure began with the collection of demographic information. Participants completed demographic information forms, and each participant’s body weight and BMI were measured using a composition analyzer (In Body 570, Cerritos, CA, USA). Subsequently, they performed the EF tasks in a quiet classroom with dim lighting. Participants were positioned 30–80 cm from the screen, adjusted for their vision and height. To minimize learning effects, participants practiced the test battery twice before formal testing. The tasks were administered in a consistent order, starting with the Flanker task to measure inhibition control (Eriksen and Eriksen, 1974) followed by the Corsi-block backward task to measure working memory (Corsi, 1972; Kessels et al., 2008), with a 2-min rest between tasks. Performance was automatically recorded by the computer.

CRF assessments were performed last. Participants completed a 10-min warm-up consisting of a 400-meter jog and stretching exercises, followed by the 20-m shuttle run version of the multi-stage fitness test on an outdoor playground to assess maximal oxygen consumption (VO2 max) for CRF evaluation. Participants continued the shuttle runs in sync with audio cues until they reached voluntary exhaustion or could no longer maintain the required pace. Assessors recorded the levels and number of shuttles for further analysis.

The post-test followed the same procedure as the pre-test, with EF tasks conducted first, followed by the CRF assessments. Daily physical activity outside of training classes was recorded (i.e., IPAQ) at last. Testing sessions were scheduled in the morning or afternoon, depending on participants’ availability.

### 2.3 Intervention

The intervention period lasted 16 weeks, during which participants in the intervention groups (golf and football) engaged in specific training sessions led by qualified coaches, while the control group continued their regular physical education classes. Each intervention group participated in four 90-min training sessions per week, while the control group attended one 80-min physical education class per week. The content of the training sessions varied according to the assigned group:

Participants in the golf group received the following training: (1) Driving range practice: focused on developing swing techniques and improving distance control. (2) Course play: practical application of skills in a real-game setting to enhance strategic thinking and course management. (3) Putting practice: aimed at refining precision and accuracy in short-distance shots. (4) Strength training: included exercises to improve overall muscular strength and endurance, which are essential for maintaining form and reducing injury risk during swings.

Participants in the football group received the following training: (1) Ball control exercises: drills designed to enhance dribbling, passing, and receiving skills. (2) Skills training: focused on specific football techniques such as kicking, dribbling, and shooting. (3) Skill combination drills: combine various skills in a single exercise to simulate real-game scenarios and improve overall gameplay. (4) Opposition drills: practiced against defenders to develop tactical awareness and decision-making under pressure. (5) Standard matches: regular gameplay to apply learned skills in a competitive environment. (6) Physical conditioning: included aerobic and anaerobic exercises to improve cardiovascular fitness, speed, and agility.

Participants in the control group engaged in general physical education activities that were less structured and of lower intensity compared to the intervention groups. These activities included: (1) General fitness exercises: such as jogging, stretching, and light aerobic activities with minor strength training. (2) Sports of individual choice: activities chosen by the participants, typically performed at low to moderate intensity levels.

During the intervention, coaches strictly adhered to the training plans to prevent injuries and ensure participant well-being. Participants were instructed to immediately report to the coach if they felt the training intensity was too high or if they experienced any discomfort or potential injury, allowing them to rest as needed. The training program was only interrupted by adverse weather conditions and public holidays. During bad weather, indoor classes focusing on the theoretical knowledge of the respective sports were conducted. Adherence rates were recorded by the coaches throughout the intervention period, resulting in 98.21% adherence in the football group, 98.59% in the golf group, and 94.64% in the control group.

### 2.4 Measurement

#### 2.4.1 Executive function

The Psytoolkit battery was used to assess EF performance (Stoet, 2010, 2017). In Flanker task (Eriksen and Eriksen, 1974), participants sat in front of a computer screen and saw five letters appearing above a fixation point (a white cross). At the start of the test, they had to respond only to the central letter, by pressing the A button on the keyboard (if the central letter was either X or C), or by pressing the L button (if the central letter was either V or B). If the flanking letters’ response does not match the response required by the central letter, we refer to it as an “incongruent” condition. Participants were given 50 trials and were asked to respond as quickly and accurately as possible. Participants underwent two blocks of 25 trials each. The 50 trials presented in this task were distributed equally among the two experimental conditions and were presented in a random order. Participants had 2 s to provide their response to the correct letter. Performance was recorded by corrected reaction time (ms) in two different conditions, as it address the speed–accuracy interactions for EF performance (Draheim et al., 2019).

In Corsi-block backward task (Corsi, 1972; Kessels et al., 2008), participants were instructed to use their computer mouse to click through a series of up to nine previously highlighted blocks in reverse order. This modification of the task not only required participants to maintain the information given but to also manipulate its order, making this version more cognitively taxing than the traditional Corsi-block task which needs to click with the same order (Kessels et al., 2008). The blue squares briefly change color to yellow in a sequence, and once the sequence is complete, an auditory (“Go”) alert notified the participant to click the squares in the reverse order they saw them change color. The blocks started with the 2 blocks, should the participant tap the given sequence correctly, they moved on to a more complicated one, with one block added. The test was discontinued when a participant incorrectly indicated the pattern twice. The participant’s scores were the highest level reached before the discontinue rule was met. For example, if a participant correctly indicated the pattern at two, three, four, and five squares, and then indicated the incorrect pattern twice at six squares, then their score on the Corsi task was five. The number of blocks was recorded for performance analysis.

#### 2.4.2 Cardiorespiratory fitness

The 20-meter shuttle run version of the multi-stage fitness test were used to assess maximal oxygen consumption (VO_2 max_) for CRF evaluation (Léger and Lambert, 1982). The starting speed was 8.5 km/h, which increased by 0.5 km/h every minute. VO_2 max_ was then estimated using an online calculator based on the equation developed by Léger et al. (1988), which was calculated by the participants’ performance data (i.e., levels and number of shuttles) from the multi-stage fitness test.

#### 2.4.3 International physical activity questionnaire

The short version of the International Physical Activity Questionnaire (IPAQ) is frequently utilized in academic research to quantify physical activity levels among populations (Lee et al., 2011; Liang et al., 2022). This survey includes 7 questions that cover various intensities of physical activity—vigorous, moderate, and walking—as well as sitting time over the last 7 days. Results from the IPAQ expressed in terms of metabolic equivalent tasks (METs) minutes per week, providing a standardized metric for comparing physical activity levels across different studies and demographic groups. Notably, for participants in the golf group, on-course training (i.e., playing 9 or 18 holes) is classified as moderate activity, while practice on hitting mats and the driving range is classified as walking (Eigendorf et al., 2020; Scalise et al., 2024).

### 2.5 Statistical analysis

Preliminary analysis was conducted. Outliers were identified using boxplots and *z*-scores, with values exceeding ±3 standard deviations (SD) considered potential outliers. However, even if a value exceeded 3 SD, it was retained in the analysis as long as it remained within the normal measurement range, to better reflect real-life data variability. Minor instances of missing data were addressed using mean substitution.

A repeated-measures analysis of variance (ANOVA) with three groups (golf, football, and control) and two time points (pretest and posttest) was conducted to examine the between- and within-condition effects on EF and CRF. EF performance was analyzed separately for the congruent and incongruent conditions of the Flanker task, as well as the Corsi-block backward task. If a significant effect was observed, *post hoc* tests with Bonferroni correction were performed. The Pearson correlation test was used to examine the association between CRF and EF performance at post-test. Effect size is presented as partial eta squared (η^2^) for ANOVA and Cohen’s d for *post hoc* comparison. The significance level was set to 0.05. Effect sizes of η^2^ and d of 0.01, 0.06, 0.14; and 0.2, 0.5, 0.8 were considered as the small, medium and large effects. Statistical analyses were performed using SPSS (version 29, IBM SPSS, Armonk, NY, USA).

## 3 Results

### 3.1 Flanker task

#### 3.1.1 Congruent

**Figure 1:**
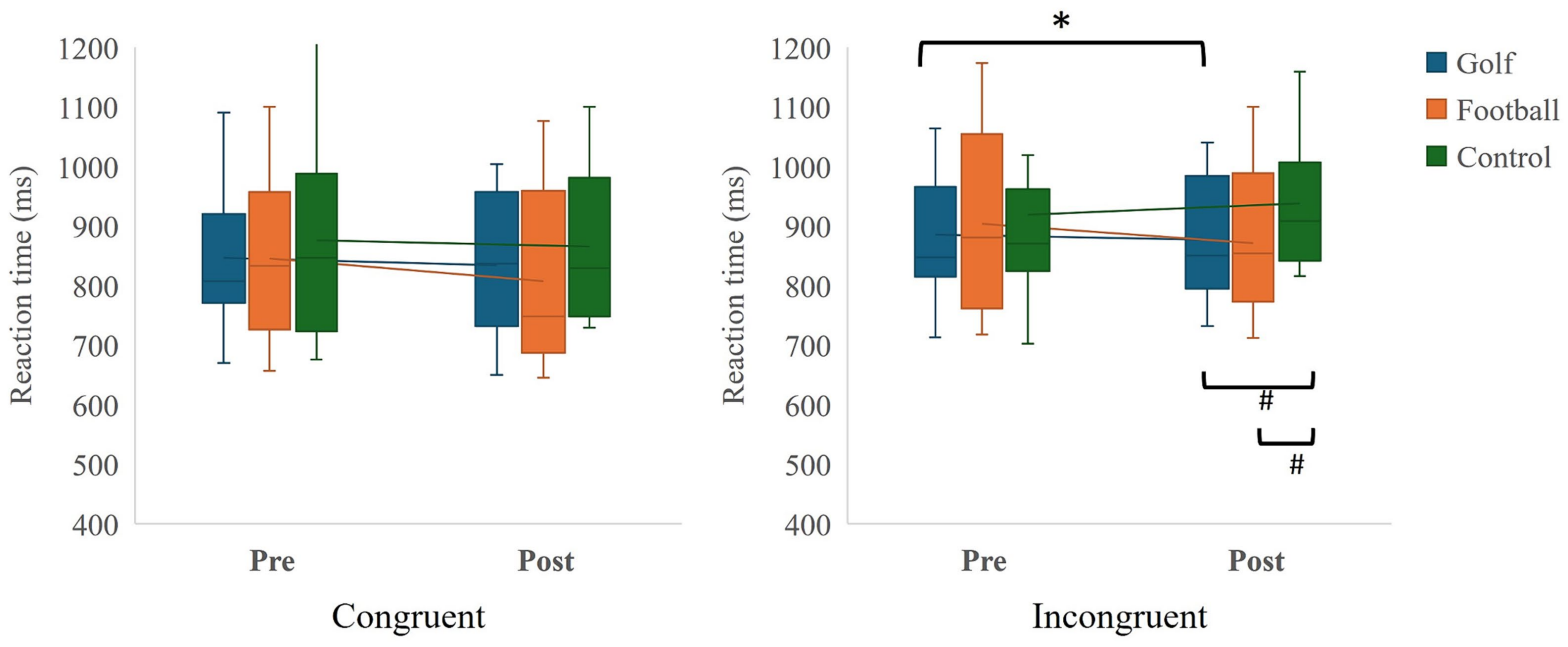
Flanker task. * Pre-post comparison with Bonferroni correction *p* < 0.05; # comparisons at post-test with Bonferroni correction *p* < 0.05.

The interaction effect between groups and time was not significant, *F*(2, 40) = 2.5, *p* = 0.10, η^2^ = 0.11. Furthermore, there were no significant main effects for either group *F*(2, 40) = 0.92, *p* = 0.41, η^2^ = 0.04, or time, *F*(1, 20) = 1.32, *p* = 0.26, η^2^ = 0.06. (see Figure 1 and Table 2).

**Table 2:** Performance of executive tasks.

**Variables**	**Golf**	**Football**	**Control**	**Golf**	**Football**	**Control**
	Pre			Post		
**FT**						
Congruent	846.38 (113.23)	845.33 (139.12)	875.48 (171.84)	814.71 (117.53)	834.43 (140.40)	893.33 (126.84)
Incongruent	885.67 (106.00)	903.90 (148.49)	918.76 (160.74)	859.14 (96.12) *^, #^	883.05 (127.24) ^#^	964.29 (115.98)
**CBBT**						
	6.43 (0.93)	6.48 (0.68)	6.43 (0.87)	6.52 (0.87)	6.81 (1.03)	6.67 (1.15)

Data are presented as mean (SD). FT indicates the Flanker task (reaction time); CBBT indicates the Corsi-block backward task (corrected numbers). *p < 0.05 compared with pre-test after Bonferroni correction. ^#^*p* < 0.05 compared with control group at post-test after Bonferroni correction.

#### 3.1.2 Incongruent

The interaction effect between groups and time was significant *F*(2, 40) = 6.97, *p* = 0.003, η^2^ = 0.26. However, neither the main effects of groups, *F*(2, 40) = 2.02, *p* = 0.15, η^2^ = 0.09, nor time, *F*(1, 20) = 0.01, *p* = 0.94, η^2^ < 0.001 were significant. *Post hoc* analysis showed a significant reduction in reaction time for the golf group from pre- to post-test (*p* = 0.018, *d* = 0.26). However, the changes in the football group (*p* = 0.09) and the control group (*p* = 0.04) were not significant after Bonferroni adjustment. At post-test, both the golf group (*p* = 0.002, *d* = 0.99) and the football group (*p* = 0.01, *d* = 0.67) performed better than the control group. However, no significant difference was found between the golf and football groups post-intervention (*p* = 0.50) (see Figure 1 and Table 2).

### 3.2 Corsi-block backward task

**Figure 2:**
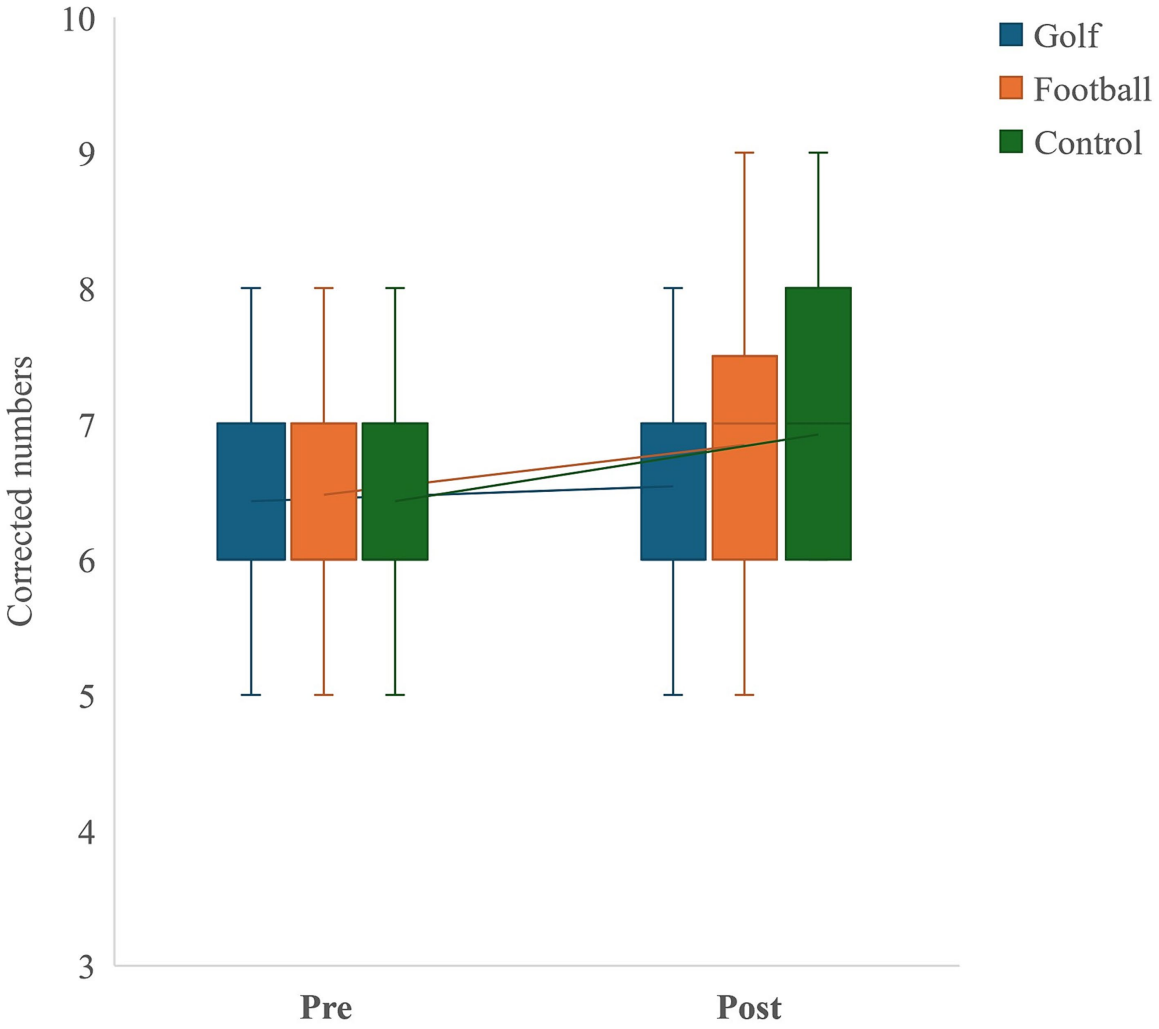
Corsi-block backward task.

The interaction effect between groups and time was not significant, *F*(2, 40) = 0.34, *p* = 0.72, η^2^ = 0.02. Furthermore, there were no significant main effects for either group, *F*(2, 40) = 0.25, *p* = 0.78, η^2^ = 0.01 or time, *F*(1, 20) = 2.80, *p* = 0.11, η^2^ = 0.12. (See Figure 2 and Table 2).

### 3.3 Cardiovascular fitness

**Figure 3:**
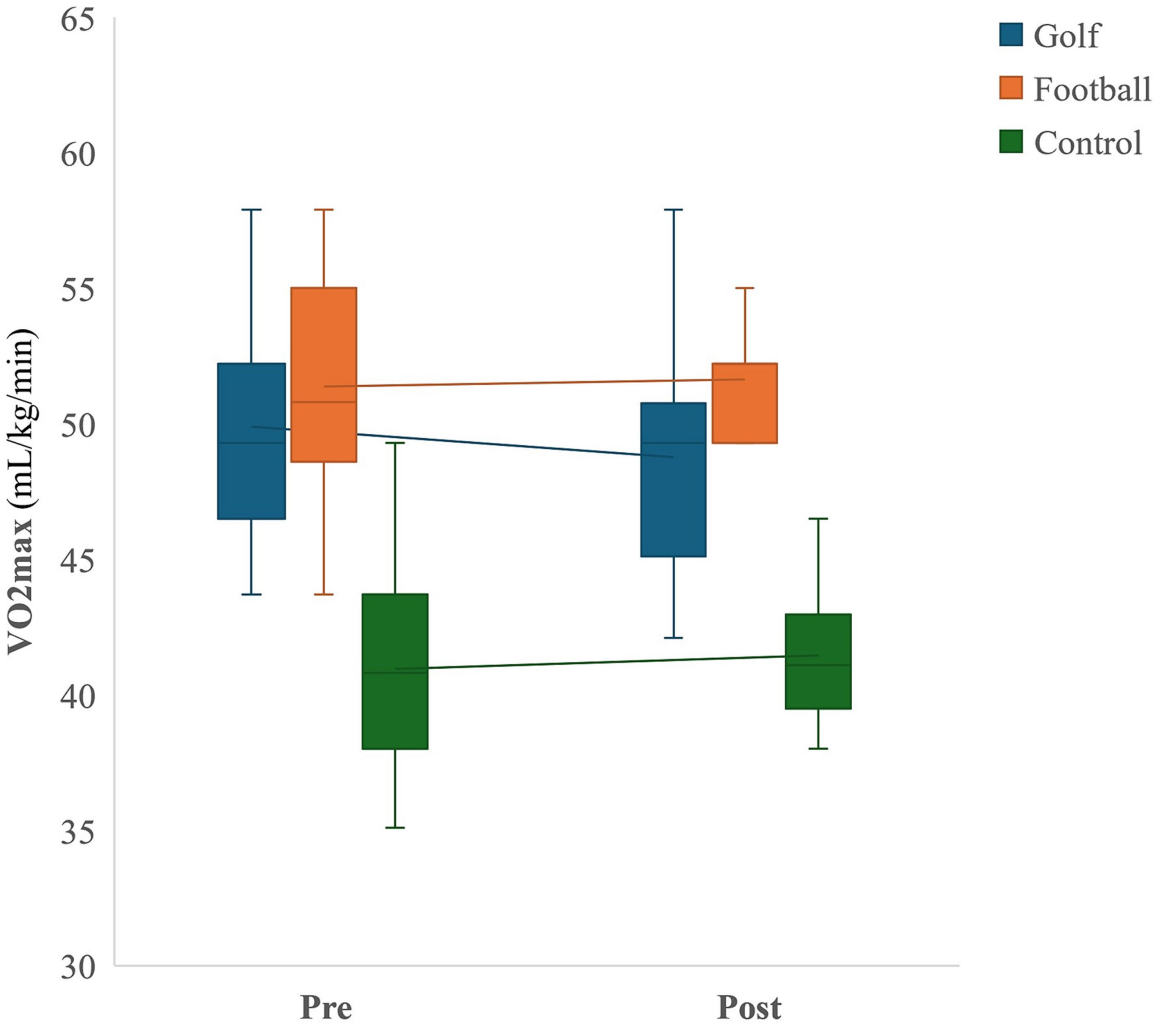
Cardiovascular fitness.

The interaction between groups and time was not significant, *F*(2, 40) = 2.86, *p* = 0.07, η^2^ = 0.13. The main effect of time was not significantly different, *F*(1, 20) = 0.22, *p* = 0.65, η^2^ = 0.01. However, there was a significant main effect of groups, *F*(2, 40) = 54.75, *p* < 0.001, η^2^ = 0.73. Pairwise comparisons with Bonferroni correction revealed that, regardless time effect, both the golf and football groups had significantly higher CRF scores compared to the control group (all *p* < 0.001) (Figure 3).

Correlation analyses indicated that there were no significant associations between VO_2max_ and EF performance on the Flanker task congruent condition (*r* = −0.16, *p* = 0.20) or incongruent condition (*r* = −0.18, *p* = 0.16). Additionally, no significant correlation was found between VO_2max_ and CBBT task (*r* = −0.30, *p* = 0.80).

## 4 Discussion

This study investigated the effects of football (open-skills) and golf (closed-skills) on executive function improvement in male university students. After 16 weeks of training, the golf group demonstrated significant improvements in inhibition control compared to their pre-training levels, while no significant improvement was observed in the football or control groups. However, the golf group did not show superior performance to the football group at post-training, although both intervention groups outperformed the control group. Notably, cardiovascular fitness did not change significantly and was not correlated with changes in EF, suggesting that the observed improvements in EF may be more closely related to the cognitive demands of the interventions rather than changes in physical fitness. These findings could inform strategies for integrating sports into educational and health interventions, ultimately contributing to improved cognitive outcomes across various populations.

The results support our hypothesis that golf, as a close-skill exercise with high cognitive demand, was effective in enhancing inhibition control after training. This finding aligns with Wang et al. (2020), who found that elite golfers exhibited stronger inhibitory control compared to amateurs, as measured by EEG, showing higher levels of attention to motor programming, visuospatial processing, and reduced cognitive-motor interference before putting. However, the precise mechanisms underlying these improvements remain unclear. One possible explanation is that the intense focus, strategic thinking, and precise motor control required in golf engage neural circuits associated with EF, particularly those governing inhibitory control. Since performing complex, multitasking tasks in stressful environments can enhance connectivity within large neurocognitive networks (Kim et al., 2017), the cognitive demands of golf may provide sufficient stimulation to produce lasting improvements in EF (Gallicchio et al., 2017; Shimada et al., 2018; Roberts et al., 2021; Wang et al., 2020). Additionally, golf training may also increase handgrip strength, which has been linked to enhanced EF in previous studies (Carson, 2018; Zhu et al., 2022). Interestingly, we observed no significant improvement in working memory between the groups, which is consistent with Lineweaver et al. (2019), who observed that frequent exercise is not associated with better working memory capacity than infrequent exercise in non-athlete college students. Similarly, Song et al. (2024) found that while there was no significant difference in working memory task performance between athletes and non-athletes at the behavioral level, although functional near-infrared spectroscopy (fNIRS) revealed differences in prefrontal cortex activation during the task.

Our finding is in contrast to previous reviews that have generally concluded that open-skill sports are more effective at enhancing EF due to their association with increased neural efficiency during cognitive tasks (Gu et al., 2019; Zhu et al., 2020; Heilmann et al., 2022; Takahashi and Grove, 2023). This discrepancy may arise because prior studies on closed-skill exercises primarily focused on activities with minimal cognitive load, such as running or swimming, potentially lacking persuasive evidence (Heilmann et al., 2022). As a result, these comparisons have not fully explored whether closed-skill exercises with high cognitive demands, like golf, can produce similar EF improvements as open-skill exercises. Our study addresses this gap in the literature by directly comparing the effects of open-skill and closed-skill sports on EF in a controlled setting. This is particularly significant as previous research has not adequately examined the role of cognitive load in determining EF outcomes across different types of sports.

Interestingly, unlike previous studies, football did not lead to significant improvements in EF in our study. This finding may be attributed to the fact that the participants had already reached sub-elite levels of physical fitness, necessitating more challenging stimuli to achieve further EF gains. It’s possible that the relationship between CRF and EF follows a curvilinear pattern, where increases in CRF beyond a certain threshold have diminishing or negligible effects on EF improvement. However, to our best knowledge, no studies have identified this relationship or established a cutoff value to delineate this threshold. This might also explain the inconsistencies observed in previous studies, where most acknowledge a correlation between CRF and EF (Mekari et al., 2019; Aghjayan et al., 2021; Logan et al., 2023), while some report no significant relationship (Tuvey et al., 2019; Meijer et al., 2021). Based on our findings, for individuals who have already achieved moderate to high levels of CRF, interventions with greater cognitive load may be more effective in stimulating and enhancing EF.

Regarding the observation that both intervention groups performed better than the control group in post-test inhibition control, this finding is consistent with mainstream research that suggests exercise can enhance EF performance in youth (Liu et al., 2020; Li et al., 2022). The athletes generally exhibit better EF compared to sedentary or non-athletic individuals, especially for attention and inhibition control capacity (Jacobson and Matthaeus, 2014; Sharma et al., 2019). This suggests that increasing physical activity may be an effective strategy for improving EF performance in sedentary students. For instance, Wu et al. (2022) found that breaking up prolonged sitting with light-intensity exercise can enhance attention, EFs, and mood. The findings highlight the importance of incorporating regular exercise into the routines of sedentary students (Zhu et al., 2024), in order to improve their EF performance and well-being.

The findings of this study have significant implications for both research and practice. Our results contribute to the growing evidence that integrating cognitive demands into exercise training can enhance EF more effectively than merely increasing exercise intensity. This aligns with studies by Biazus-Sehn et al. (2020) and Gavelin et al. (2021), which demonstrate that combining cognitive challenges with physical activity leads to superior cognition improvements. Additionally, our findings suggest that for individuals with moderate to high levels of physical fitness, engaging directly in cognitively demanding activities appears to be an effective targeted intervention for enhancing EF. This is further supported by Casella et al. (2022), who found that a 10-week cognitive-motor training program integrated into standard football training significantly improved planning abilities and visual search in young football players. Similarly, Kolovelonis and Goudas (2023) found that primary students involved in cognitively challenging games showed greater improvements in EF performance compared to those in football, track and field, and control groups after 1 month of intervention.

This study has several limitations. First, the effect size for the improvement observed in the golf group from pre- to post-test is relatively small, the result should be interpreted with caution. Second, only male college athletes were recruited, which limits the generalizability of our findings to other populations. Third, this study employed convenience sampling, which may have introduced bias into the statistical results (Duan et al., 2022). Future studies are recommended to use random sampling to strengthen the findings. Fourth, our research focused on behavioral outcomes; due to experimental constraints, we did not use neuroimaging techniques (e.g., EEG, fNIRS) or measure the biomarkers (e.g., brain-derived neurotrophic factors and catecholamines) which relatively with EF changes.

## 5 Conclusion

This study provides preliminary evidence that golf, a closed-skill sport with high cognitive demands, can effectively improve inhibitory control after 16 weeks of training. While this improvement in EF is comparable to the gains observed in football, an open-skill sport, it underscores the cognitive health benefits that closed-skill sports may provide. Further research is needed to explore the underlying neural mechanisms and to determine whether similar improvements can be achieved in broader populations.

## Data Availability

The datasets used and/or analyzed in the current study are available from the corresponding author upon reasonable request.
